# 节拍化疗——晚期非小细胞肺癌另辟蹊径的治疗策略

**DOI:** 10.3779/j.issn.1009-3419.2015.04.08

**Published:** 2015-04-20

**Authors:** 爽 张, 菁菁 柳, 颖 程

**Affiliations:** 130012 长春，吉林省肿瘤医院胸部肿瘤内科 Department of Toracic Oncology, Jilin Provincial Cancer Hospital, Changchun 130012, China

**Keywords:** 肺肿瘤, 节拍化疗, 传统化疗, Lung neoplsms, Metronomic chemotherapy, Conventional chemotherapy

## Abstract

节拍化疗是近年来兴起的一种新的化疗策略。与传统化疗不同，节拍化疗通过相对低剂量的、频繁的应用细胞毒性药物，没有较长的治疗间歇，发挥抗肿瘤作用。最初认为节拍化疗直接作用于肿瘤血管内皮细胞，发挥抗血管生成作用。近年来发现节拍化疗还有调节机体免疫功能，影响肿瘤干细胞，诱导细胞休眠的作用。晚期非小细胞肺癌（non-small cell lung cancer, NSCLC）的治疗已经从彻底的清除肿瘤细胞转向改善疗效、降低毒性和提高生活质量。节拍化疗可以避免传统化疗毒性大，作用不持久的缺点，目前一些临床研究正在探索节拍化疗对晚期NSCLC的作用，并且初见疗效，有望成为晚期NSCLC一种新的治疗模式。

## Abstract (en)

Metronomic chemotherapy is an emerging strategy to fight cancer. Unlike traditional chemotherapy, metronomic chemotherapy is defined by the frequent, repetitive administration of chemotherapeutic drugs at relatively low doses, and without prolonged drug-free break. Initially thought to play a role inhibiting tumor angiogenesis by targeting activated endothelial cells in tumors, metronomic chemotherapy is a multi-targeted therapy, including activation of immunity, effect on tumour initiating cells, induction of tumor dormancy. It is from eradicateing tumor cells to improve effect, reduce the toxicity and improve quality of life for treatment of advanced non-small cell lung cancer (NSCLC). Metronomic chemotherapy which can avoid the toxicity of traditional chemotherapy and rebounding is explored in clinical studies of advanced NSCLC, as a promising treatment strategy.

化疗是晚期非小细胞肺癌（non-small cell lung cancer, NSCLC）治疗的基石，然而传统化疗采用最大耐受剂量（maximum tolerated dose, MTD），毒性大，作用不持久，容易耐药，限制了临床应用。2000年Folkman及其同事^[1]^通过动物移植瘤模型发现持续应用小剂量环磷酰胺能够使肿瘤血管床的内皮细胞持续凋亡，同年Kerbel^[2]^也证实持续低剂量的长春花碱能够抑制肿瘤血管生成，使移植瘤缩小。在这两项开创性的研究基础上，Hanahan^[3]^提出了节拍化疗（metronomic chemotherapy）的概念。

节拍化疗是规律的、频繁的给予低剂量的细胞毒药物（1/10-1/3的MTD），而没有较长的治疗间歇^[3, 4]^。与传统化疗针对快速增殖的肿瘤细胞不同，节拍化疗主要作用于血管内皮细胞和循环内皮祖细胞（circulating endothelial progenitors, CEPs）。研究^[5]^发现血管内皮细胞对紫杉类药物的敏感性是HT1080人纤维肉瘤细胞的10倍-100倍，提示血管内皮细胞较肿瘤细胞对细胞毒药物更敏感。分别用传统化疗和节拍化疗处理鼠胰腺癌移植瘤^[6]^，节拍化疗使CD31标记的血管内皮细胞显著减少，而传统化疗对其无影响，表明节拍化疗直接抑制血管内皮细胞的增殖。CEPs来源于骨髓，是血管内皮细胞的前体，CEPs释放到外周血被认为是加速肿瘤生长和发生耐药的关键步骤^[7]^。而传统化疗和节拍化疗对CEPs的影响是截然不同的，鼠淋巴瘤移植瘤模型中，传统化疗后出现CEPs反弹，肿瘤迅速生长和耐药，而节拍化疗后CEPs数量持续减少，活性下降，并出现持久的肿瘤生长抑制^[8]^。传统化疗在给予患者MTD后，有一个较长的间歇期，使患者能够从治疗的相关毒性中恢复，与此同时肿瘤细胞通过动员CEPs促进肿瘤血管生成使肿瘤重新生长^[8-11]^。节拍化疗直接靶向内皮细胞，避免血管生成反弹，同时降低了治疗相关毒性^[12]^。另外肿瘤与所处的微环境相互作用，传统化疗仅针对快速增殖的肿瘤细胞，无法根除肿瘤^[13-15]^，而且传统化疗杀死化疗敏感的肿瘤细胞，留下化疗耐药的肿瘤细胞，而产生获得性耐药，最终导致疾病复发。节拍化疗同时针对敏感和耐药肿瘤细胞，既针对肿瘤细胞，也针对肿瘤微环境中的内皮细胞和其他的支持细胞，极少产生获得性耐药^[14]^。随着研究的进展，人们对节拍化疗的认识不断深入，从最初认为节拍化疗主要通过抑制血管生成发挥抗肿瘤作用，到现在发现节拍化疗通过对肿瘤细胞及其肿瘤微环境的影响而发挥多方面的抗肿瘤作用。

## 1 节拍化疗的抗肿瘤血管生成作用

节拍化疗抗血管生成的理论来源于Folkman和Kerbel动物实验。新形成血管床内活化的内皮细胞对一些低剂量的化疗药物是高度敏感的^[16, 17]^。节拍化疗能够抑制内皮细胞的迁移，影响肿瘤血管内皮细胞增殖，诱导活化的内皮细胞凋亡，促进内源性抗血管生成因子血小板反应蛋白-1（thrombospondin 1, TSP-1）的表达^[18]^。

在漫长的肿瘤血管生成的过程中，肿瘤血管内皮细胞也会出现增殖状态，但与肿瘤细胞相比，增殖的频率较低。传统化疗对低频率增殖、分化的血管内皮细胞只有微弱的破坏作用，低剂量持续应用细胞毒性化疗药物可以作用于缓慢增殖的肿瘤血管内皮细胞，对治疗间歇期血管内皮细胞的修复能力造成损害。Kerbel^[16]^为了证实与其他细胞相比活化的内皮细胞对低剂量的节拍化疗更为敏感，评价了人血管内皮细胞、成纤维细胞、敏感的乳腺癌细胞和耐药的乳腺癌细胞短期（24 h）和长期暴露（144 h）不同的化疗药物对细胞的影响，发现短期暴露对各种细胞的影响几乎没有差别或者只有微弱的影响，而长期持续暴露细胞毒性药物，内皮细胞相对高度敏感，呈现皱缩趋势。而且半数抑制浓度（half growth inhibition concentration, IC_50_）在25 pm^-1^43 pm时能够出现内皮细胞生长抑制，还能够诱导凋亡；而抑制肿瘤细胞和成纤维细胞生长的IC_50_浓度较高，在500 pm^-1^ nm。

TSP-1是细胞外基质成分之一，为内源性血管生成抑制因子，与表达在内皮细胞上的分化抗原36（cluster of differentiation 36, CD36）受体结合，抑制内皮细胞增殖，促进凋亡。Kalluri^[19]^研究发现低剂量的环磷酰胺治疗鼠移植瘤模型显著提高TSP-1的表达。体外研究^[20]^发现内皮细胞持续低剂量暴露于多种细胞毒性药物，能够在基因水平和蛋白水平提高TSP-1的表达。

研究发现节拍化疗还通过减少CEPs的数量和活性而抑制肿瘤血管生成。Kerbel^[8]^通过免疫缺陷鼠淋巴瘤模型，发现最大耐受剂量的环磷酰胺治疗后出现强烈的CEPs抑制，但很快出现耐药，而环磷酰胺节拍治疗使CEPs数量减少和活性的下降与更长时间的抑制肿瘤生长一致。提示低剂量节拍化疗方案是避免CEPs动员的方案。

## 2 节拍化疗对免疫功能的调节

逃逸机体的免疫监视已经成为癌症的特征之一。先天性和获得性免疫系统在控制肿瘤发生和发展的过程中均发挥重要的作用。而化疗对机体免疫系统的影响是复杂多样的。传统化疗通常发挥免疫抑制作用，然而有研究发现一些化疗药物以特殊方式应用如节拍化疗表现为有助于肿瘤根除的积极的免疫作用^[16, 21]^。节拍化疗能够使肿瘤患者体内的免疫平衡由免疫抑制状态向免疫活化状态转换^[9]^。

调节性T细胞（regulatory t cells, Treg）是CD4^+^CD25^+^ Foxp3^+^淋巴细胞，表达细胞毒性淋巴细胞相关抗原-4（cytotoxict-lymphcyte-associated antigen-4, CTLA-4），能够抑制细胞特异性免疫应答。Treg也能抑制由CD8^+^淋巴细胞、CD4^+^T辅助细胞和自然杀伤细胞（natural killer cell, NK）介导的抗肿瘤免疫应答^[22, 23]^。Treg的增加与肿瘤进展及对抗肿瘤治疗缺少应答有关。因此清除肿瘤患者的Treg，尤其是肿瘤微环境中的Treg，被认为是成功的抗肿瘤治疗的重要组成部分之一^[23]^。近年来，一些研究发现在动物移植瘤模型中，节拍化疗能够通过减少Treg细胞数量和抑制Treg功能，增加抗肿瘤免疫反应。Banissi等^[24]^通过不同的替莫唑胺方案处理替莫唑胺耐药的鼠胶质瘤模型，观察对Treg数量的影响，结果发现只有替莫唑胺节拍治疗方案能够减少Treg/CD4^+^的比例，而且由节拍化疗诱导的Treg的消耗同时还伴随着残存Treg免疫抑制功能的下降。Ghiringhelli等^[25]^也证实环磷酰胺节拍化疗治疗晚期肿瘤患者也能够明显减少循环中的Treg。

髓样抑制细胞（myeloid-derived suppressor cells, MDSC）是另一种发挥肿瘤免疫抑制作用的细胞成分，在许多实体瘤中能够抑制T细胞的活性^[26]^。研究发现节拍化疗在选择性清除MDSC的同时还有保护具有潜在的增强抗肿瘤免疫的T细胞亚群的作用^[26]^。一项体内研究^[27]^发现紫杉醇节拍化疗能够减少肿瘤组织内浸润的MDSC，而对骨髓造血干细胞没有影响，同时还发现MDSC的减少与抑制p38丝裂原活化蛋白激酶（mitogen-activated protein kinase, MAPK）的活性，减少肿瘤坏死因子-α（tumor necrosis factor-α, TNF-α）和一氧化氮（nitric oxide, NO）产生，抑制S100A9的表达有关。Vincent研究^[28]^发现氟尿嘧啶节拍化疗治疗鼠肿瘤模型，使脾脏和瘤床中MDSC减少，而对B细胞、NK细胞、树突状细胞（dendritic cells, DC）、T细胞没有影响。在清除MDSC和诱导MDSC凋亡中，氟尿嘧啶节拍化疗较吉西他滨节拍化疗作用更强，研究发现5-氟尿嘧啶通过增加肿瘤组织中CD8^+^T细胞产生干扰素-γ（interferon-γ, IFN-γ），进而促进T细胞依赖的抗肿瘤免疫应答。

DC是主要的抗原提呈细胞，临床前和临床研究发现以DC为基础的免疫治疗能够诱导强烈的抗肿瘤免疫应答^[29]^。研究发现节拍化疗能够诱导DC成熟，增强DC的吞噬作用，而且能够活化DC上的Rho家族的鸟苷三磷酸酶（Rho family of guanosine triphosphate phosphatase, Rho GTPases），Rho GTPases在调节细胞之间的相互作用、细胞运动、内吞作用中发挥重要作用^[30]^。

此外节拍化疗还通过增强肿瘤的免疫原性、提高免疫效应细胞如肿瘤特异性T细胞^[31]^和γδT细胞^[32]^的细胞毒活性，刺激钙网蛋白和热休克蛋白的暴露，促进内源性危险信号的释放^[21]^，而发挥促进抗肿瘤免疫的作用。

## 3 节拍化疗对肿瘤干细胞的影响

肿瘤干细胞（cancer stem cells, CSC）是肿瘤内具有持续自我更新、无限增殖的，驱动肿瘤不断进展的一群细胞，是对传统化疗耐药的根源。因此需要新的策略清除CSCs，同时对正常的干细胞没有影响。

越来越多的证据显示节拍化疗也直接作用于肿瘤细胞，包括CSCs。Folkins等^[33]^的研究发现环磷酰胺节拍化疗能够显著减少原发的和转移的肿瘤的形成。而且在胰腺癌移植瘤模型发现环磷酰胺节拍化疗治疗能够减少CD133^+^前体细胞和CD133^+^/CD44^+^/CD24^+^肿瘤干细胞的数量^[34]^。鼠移植瘤模型中，节拍化疗与直接的抗血管药物联合能够有效的减少CSCs的数量^[33]^。体外研究发现长期暴露于紫杉醇或者依托泊苷的前列腺癌细胞和结肠癌细胞通过表观遗传学机制会产生耐药细胞系，有趣的是，在体内研究发现这些耐药细胞系的致瘤性却下降，与亲代细胞相比CD44^+^细胞群明显减少，提示节拍化疗可能靶向CD44^+^的CSCs^[35]^。

## 4 节拍化疗诱导肿瘤休眠

既往研究^[36, 37]^认为肿瘤细胞休眠在抑制肿瘤进展和复发中发挥重要作用。肿瘤细胞休眠是细胞周期阻滞的结果，同时也是肿瘤细胞增殖和凋亡平衡的结果。肿瘤休眠可以发生在原发肿瘤部位，也发生在肿瘤转移部位，通常分为三种不同的类型：血管生成休眠、细胞休眠和免疫监视^[15]^。最初Folkman等^[38]^的研究认为无血管的肿瘤处于休眠状态，而且在视网膜母细胞瘤中也可以观察到这种肿瘤休眠状态^[39]^，因此节拍化疗通过抑制肿瘤血管生成也可能诱导和维持血管休眠状态，而且在节拍化疗中涉及的血管休眠机制也可能与其对免疫系统的影响产生叠加作用。免疫系统参与控制和根除肿瘤，肿瘤特异性细胞毒性T淋巴细胞能够诱导肿瘤细胞凋亡，残存的肿瘤细胞通常持续存在，但通过免疫系统使其保持休眠状态，休眠状态的中断可能是发生了免疫逃逸^[40]^，因此通过节拍化疗加强抗肿瘤免疫是促进肿瘤休眠的一种机制。但是目前仍然没有直接的证据支持节拍化疗促进肿瘤休眠。

肿瘤的发生发展是与所处的微环境相互作用的复杂的过程，节拍化疗不但能够直接影响肿瘤细胞、也作用于肿瘤所处的微环境，影响肿瘤血管生成和免疫系统，而且还通过三者直接的相互影响增强抗肿瘤作用。

## 5 节拍化疗在肺癌中的临床应用

节拍化疗作为一种安全、有效，能够克服肿瘤耐药的一种新的治疗策略，在各种实体瘤进行大量的研究。目前节拍化疗主要用于复发耐药的晚期肿瘤。最近Lien等^[41]^对节拍化疗进行了系统性回顾分析，研究纳入3, 700例患者，共80项小样本的单臂研究及Ⅱ期研究，其中包括乳腺癌研究21项（26.25%），前列腺癌研究9项（11.25%），肺癌7项（8.75%），涉及的节拍化疗药物主要包括环磷酰胺46项（43.0%），卡培他滨15项（14.0%），足叶乙甙15项（14.0%），长春瑞滨15项（14.0%），客观缓解率（objective response rate, ORR）为26.03%，中位疾病控制率（disease control rate, DCR）为56.3%，中位无进展生存时间（progression-free survival, PFS）为4.6个月，3级/4级毒性罕见。研究认为节拍化疗对多种肿瘤是有效的，而且是安全可行的。

目前推荐的晚期NSCLC的二线用药只有多西他赛、培美曲塞、厄洛替尼，三线治疗只有厄洛替尼被推荐，三线以上治疗仍然没有推荐。二线以上化疗的ORR不足10%，PFS约2.5个月，总生存期（overall survival, OS）约9个月^[42]^。节拍化疗多作为晚期NSCLC的二线或二线以上治疗。采用的形式包括单药节拍化疗，两种或两种以上化疗药物组合的节拍化疗，节拍化疗与抗血管药物联合治疗以及节拍化疗和酪氨酸激酶抑制剂（tyrosine kinase inhibitor, TKI）靶向药物联合。Kontopodis等^[43]^进行了一项节拍化疗挽救性治疗晚期NSCLC的Ⅱ期研究，46例患者接受长春瑞滨口服50 mg/次，每周3次，28 d为1个周期，直至疾病进展或者毒性不能耐受，其中5例患者部分缓解（partial response, PR），9例疾病稳定（stable disease, SD），DCR为30.4%，而在长春瑞滨静点和口服制剂传统方案挽救性治疗晚期NSCLC的两项Ⅱ期研究中都没有观察到客观缓解^[44, 45]^。在每周紫杉醇节拍化疗治疗晚期转移性NSCLC的回顾性研究^[46]^中，100例经治的晚期NSCLC或者复发的NSCLC以及一般状态较差的患者，接受每周紫杉醇治疗，ORR为37%，35%的患者SD，3级毒性包括贫血（2%），中性粒细胞减少（10%），粒缺性发热（2%），感觉神经病变（15%），运动神经病变（4%）。中位OS为12个月，中位PFS为5个月。可见每周紫杉醇治疗晚期NSCLC是有前景的方案。体外研究发现替莫唑胺对NSCLC具有抗肿瘤活性，而且对存在脑转移的晚期实体瘤包括NSCLC也具有良好的治疗作用。一项Ⅱ期研究^[47]^评价了替莫唑胺节拍化疗对经治晚期NSCLC的疗效，研究纳入31例患者，其中12例存在脑转移，28例接受过2线以上治疗，结果2例患者PR，13例获得SD，中位至疾病进展时间（time to progression, TTP）为2.4个月，中位OS为3.3个月，1年生存率22.5%，1例因4级中性粒细胞减少死亡，分别有4例和2例患者发生3级、4级淋巴细胞减少，非血液学毒性轻微，研究认为低剂量的替莫唑胺作为挽救治疗是有活性的，但需要进一步探讨与其他抗肿瘤药物联合是否能够进一步提高疗效。多西他赛是NSCLC常用的二线治疗药物，研究者也尝试了多西他赛节拍化疗的疗效和安全性。多西他赛每周节拍治疗方案和3周传统方案的Ⅱ期^[48]^和Ⅲ期^[49]^研究发现对于经治晚期NSCLC疗效是相似的，但是在不良反应方面，每周节拍治疗方案组发热性粒细胞减少发生率明显低于3周传统方案组，因此对于有发生严重粒细胞减少风险的患者每周多西他赛方案是较好的选择。

在联合节拍化疗方面，一项开放性、单臂研究^[50]^评价了环磷酰胺、足叶乙甙胶囊节拍化疗对晚期NSCLC的疗效，研究纳入33例患者（26例难治患者，7例初治患者），其中26.5%的患者疾病稳定，东部肿瘤协作组体能状态（Eastern Cooperative Oncology Group performance status, ECOG PS）评分为1分患者的PFS和OS优于ECOG PS评分为2分的患者。一项Ⅱ期研究^[51]^探讨了每周顺铂联合每天足叶乙甙节拍治疗晚期NSCLC的毒性和疗效，共纳入31例患者，ECOG PS≤3分，其中18例为Ⅲb，13例为Ⅳ期，最常见的毒性是3级中性粒细胞减少和贫血，OR为45.2%，其中两例完全缓解（complete response, CR），12例PR，DCR为58.1%，中位的TTP和OS分别为9个月和13个月。药理学分析显示，与传统方案相比，节拍方案顺铂和足叶乙甙每月的曲线下面积（area under the curve, AUC）更大，同时检测外周血血管内皮生长因子（vascular endothelial growth factor, VEGF）发现，接受节拍化疗的患者中有10例患者VEGF水平下降，而接受传统化疗的患者中有10例VEGF水平升高，提示节拍化疗通过影响VEGF水平影响肿瘤生长和肿瘤新生血管生成。研究认为节拍化疗具有较好的耐受性和活性，甚至是对一般状态较差的患者仍然适用。在多西他赛和曲磷胺节拍化疗二线治疗转移性晚期NSCLC的探索性研究中^[52]^，21例患者入组，ORR为19%，中位OS为6.9个月，中位PFS为2.9个月，1年生存率为28.6%，2年生存率为7.1%，没有4级毒性发生。研究认为多西他赛联合曲磷胺节拍化疗是NSCLC二线治疗有效的方案，具有良好的耐受性，节拍化疗有望成为NSCLC二线治疗的有效选择之一，但需要通过Ⅲ期研究进一步验证。替加氟由于具有潜在的抗肿瘤血管生成的口服药物，更适合作为节拍化疗的选择^[53]^。一项来自台湾的Ⅱ期研究^[54]^，比较了吉非替尼（250 mg/d）联合替加氟节拍化疗（1粒/次，2次/d）或者吉非替尼单药治疗既往化疗失败的晚期肺腺癌患者的疗效，58例患者接受吉非替尼单药治疗（G组），57例患者接受吉非替尼联合替加氟治疗（GU组），1年生存率分别为18%和36.7%（*P*=0.03）。54例患者提供了组织标本进行了表皮生长因子受体（epidermal growth factor receptor, *EGFR*）基因检测，G组：16例敏感突变，8例野生型，GU组：20例敏感突变，10例野生型，GU能够改善*EGFR*突变患者的PFS（14.4 mo *vs* 7.6 mo, *P*=0.006, 1）。43例患者进行了肿瘤组织微血管密度检测，发现肿瘤组织微血管密度低的患者接受GU治疗有改善PFS的趋势（11.8 mo *vs* 2.8 mo, *P*=0.053, 6)。G组和GU组的OS没有统计学差异，分别为18.3个月和23.6个月（*P*=0.381）。

临床前研究发现节拍化疗联合抗血管生成药物能够进一步提高疗效。一项研究探索了顺铂、口服足叶乙甙节拍化疗联合贝伐珠单抗治疗晚期NSCLC，评价节拍化疗是否能够增加抗血管治疗的疗效^[55]^，纳入42例患者，研究发现，联合治疗显著降低原发肿瘤的血流灌注，降低血清VEGF、血管生成素-1（angiopoietin-1）、TSP-1的水平，其中40例接受贝伐珠单抗治疗的患者中ORR为77.5%，疾病稳定率为15%，中位TTP为7.6个月，最常见的毒性为1级-2级血液学毒性，研究确定了贝伐珠单抗最佳生物剂量和最大耐受剂量分别为5 mg/kg和7.5 mg/kg。认为贝伐珠单抗联合节拍化疗是安全可行的，而且也显示了显著的抗肿瘤血管生成活性和抗肿瘤效应。在Ⅱ期研究^[56]^中进一步评价了晚期NSCLC患者接受该方案治疗的疗效，即顺铂（30 mg/m^2^, d1-3），口服足叶乙甙（50 mg, d1-d15），联合贝伐珠单抗（5 mg/kg, d3），3周为1周期，疾病没有进展的患者接受贝伐珠单抗联合厄洛替尼维持治疗，31例患者（68.8%）获得了PR，PFS为9.53个月，最常见的毒性为1/2级的血液学毒性、粘膜炎和脱发。节拍化疗联合贝伐珠单抗治疗晚期非鳞NSCLC的Ⅱ期研究^[57]^，纳入33例初治的Ⅳ期非鳞NSCLC，接受紫杉醇（80 mg/m^2^, d1, d8, d15）、吉西他滨（G）（200 mg/m^2^-300 mg/m^2^, d1, d8, d15）和贝伐珠单抗（10 mg/m^2^, d1, d15）治疗，28 d为1个周期，共6周期，没有进展的患者接受贝伐珠单抗维持治疗。1例获得CR，23例PR，6例SD，中位PFS为9个月，OS为30个月，1年生存率74%，2年生存率为55%。1例患者发生3级中性粒细胞减少，1例患者发生3级-4级恶心呕吐，2例患者3级-4级乏力，1例发生缺血性结肠炎，1例患者发生脑缺血，2例患者发生3级-4级肺炎，3例患者发生3级-4级蛋白尿没有3级-4级的高血压发生，研究认为节拍化疗与抗血管药物联合能够增强抗血管作用和增加晚期NSCLC的临床获益，但是由于入组患者有限，仍需经大样本的随机Ⅲ期研究证实。

维持治疗能够改善晚期NSCLC的PFS和OS。基于节拍化疗低毒高效，克服耐药的特点，与其他药物包括靶向治疗联合也是晚期NSCLC维持治疗的选择。目前已有两项Ⅲ期研究^[58, 59]^证实节拍化疗维持治疗是希望的治疗策略。另外研究发现节拍化疗可以和传统化疗整合，称为“化疗转换”即传统化疗后给予较长的节拍化疗维持治疗，晚期癌症患者经过传统化疗后肿瘤负荷降低，节拍化疗抑制肿瘤血管生成和调整机体免疫的作用在维持治疗阶段能够更好的发挥作用^[60, 61]^。Vives等^[34]^在胰腺癌肿瘤模型中发现传统化疗与节拍化疗转换方案较传统化疗和节拍化疗更有效。而转换方案在NSCLC中仍需进行基础和临床研究进行验证。

多数临床研究证实节拍化疗具有良好的耐受性，毒性轻微，3级毒性非常少见，常见的3级毒性包括恶心呕吐、疲乏、中性粒细胞减少、血小板减少、淋巴细胞减少。节拍化疗联合靶向治疗如贝伐珠单抗、舒尼替尼联合时观察到更严重的毒性，包括4级的咯血、血栓栓塞、肺栓塞^[55]^。另外需要关注的是节拍化疗长期应用的累积毒性和长期毒性，尤其是儿童。目前有报道^[62, 63]^替莫唑胺和依托泊苷节拍治疗有发生骨髓发育异常和继发白血病的风险。尽管生理的血管生成机制与肿瘤血管生成不同，由于血管生成在生长发育过程中发挥重要作用，对于接受节拍化疗的少年儿童患者需要特别关注。

## 5 结语

节拍化疗具有多重抗肿瘤活性，并可以与分子靶向治疗、免疫治疗联合。但是作为一种新的治疗模式，节拍化疗也面临诸多挑战。目前节拍化疗的方案多为经验性的，未来的研究需要探索适合进行节拍化疗的最佳药物，每种药物单独或联合进行节拍化疗合理的剂量、应用的频率、持续的期限，以及明确适合进行节拍化疗的人群。另外，对节拍化疗如何进行疗效监测和评价，是通过血管正常化、CEPs数量、TSP-1的水平还是通过免疫细胞数目的变化也需要大量的研究证实。
